# A Rare yet Morbid Complication of Cocaine Use: Brugada Type 1 on Electrocardiogram

**DOI:** 10.7759/cureus.24309

**Published:** 2022-04-20

**Authors:** Muhammad Atif Masood Noori, Hardik Fichadiya, Shruti Jesani, Fareeha Abid, Nikhita Sachdeva, Hasham Saeed, Qirat Jawed, Sherif Elkattawy, Meherwan Joshi

**Affiliations:** 1 Internal Medicine, Dow University of Health Sciences, Karachi, PAK; 2 Internal Medicine, Rutgers Health/Trinitas Regional Medical Center, Elizabeth, USA; 3 Internal Medicine, St. George's University School of Medicine, Union, USA; 4 Cardiology, Rutgers Health/Trinitas Regional Medical Center, Elizabeth, USA

**Keywords:** sodium channel blocker, ecg patterns, antiarrhythmic agent, cocaine, brugada syndrome

## Abstract

Cocaine is considered a leading non-opioid cause of drug overdose in the US. It acts as a sympathomimetic and increases the amount of catecholamines, thereby increasing the risk of ventricular irritability and resultant arrhythmias. Its sodium (Na) channel blockage is the principal mechanism behind the Brugada pattern on an electrocardiogram (ECG), which is often transient but is indistinguishable from that of Brugada syndrome, the autosomal dominant channelopathy.

We are presenting a case of a 32-year-old male with a history of cocaine and nicotine abuse, who sought medical attention for sudden-onset palpitations and pressure-like chest pain after having snorted an impressive amount of cocaine. His ECG depicted a classical Brugada pattern with ST-elevation with T inversion in V1; however, previous ECGs were normal without ST changes, signifying the Brugada pattern unmasked by cocaine use. Other investigations including stress tests and nuclear imaging were equivocal. His symptoms as well as the ECG pattern reverted to baseline signifying the presence of Brugada phenotype in the absence of channelopathy.

Hence, as a sodium channel blocker, cocaine may unmask latent Brugada syndrome in asymptomatic patients without a family history. Recognizing Brugada syndrome on ECG is vital to avoid misdiagnosis and mistreatment of the patient with and without a genetic predisposition.

## Introduction

Cocaine is considered a leading non-opioid cause of drug overdose in the US [1] and has gained itself a reputation as a fashionable social drug, with most of the users being young males [2]. Its decremental effects on general, as well as cardiovascular health, have been explored in-depth [3]. Cocaine-induced arrhythmias can range from benign tachycardia to the most sinister arrhythmias, from ventricular fibrillation and tachycardia to Torsade de Pointes. It causes a dose-dependent increase in heart rate and carries fast and slow sodium (Na) and potassium (K) channel blocking activities [4, 5].

Where Brugada syndrome itself is an extremely rare cardiac arrhythmia, affecting 5 in 10,000, it is classically a channelopathy [6] that is characterized by an autosomal dominant sodium channel gene mutation in SCN5A. These mutations are found in only 30% of cases and the affected individual demonstrates variable expressivity and reduced penetrance. Its phenocopy on the other hand, although having the same electrocardiogram (ECG) changes, albeit reversible, has various aetiologies including hyperkalemia and cocaine use, but without the structural peculiarities [7]. Accelerated inactivation of Na channels and predominance of transient outward K current, is the principal mechanism behind Brugada syndrome. Cocaine can lead to this erratic rhythm, given its strong* IC*, “flecainide-like” properties.

Here we present a case of a 32-year-old male with a history of cocaine abuse, who presented with acute-onset palpitations and sub-sternal pressure-like chest pain after snorting cocaine. His surface electrocardiogram depicted a characteristic Brugada picture, changed from his baseline ECG. Our case depicts the strong correlation between this substance and Brugada phenotype.

## Case presentation

We report a case of a 32-year-old male with a history of cocaine abuse and nicotine dependence who was brought to the emergency department (ED) with complaints of 3-4 hours of palpitations and anxiety that started a few hours after snorting 1.5 g of cocaine. The patient also complained of non-radiating, substernal chest pain associated with shortness of breath that resolved on its own before arrival to ED. He denied cough, fever, chills, leg swelling, dizziness and numbness or weakness in the body. He reported frequent use of cocaine with multiple histories of similar symptoms after its use. The patient has a family history of coronary artery disease (CAD).

**Figure 1:**
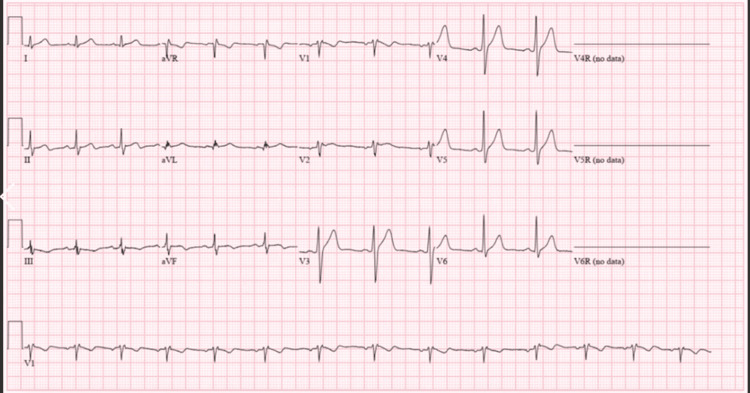
Coved-shaped ST-segment elevation with T wave inversion in lead 1

On presentation, his vitals were stable with temperature 97.2°F, blood pressure 127/88 mmHg, pulse 93/min, respiratory rate 15/min, and oxygen saturation 98% on room air. His physical examination was unremarkable. On initial labs, point-of-care troponin was elevated to 0.15 ng/ml, but subsequent troponins were within normal limits. Urine toxicology was positive for cocaine. Chest X-ray was unremarkable. ECG revealed a coved pattern of ST elevation with T inversion in V1 (Fig. 1); however, the previous ECGs were normal without ST changes, signifying the Brugada pattern unmasked by cocaine use.

**Figure 2:**
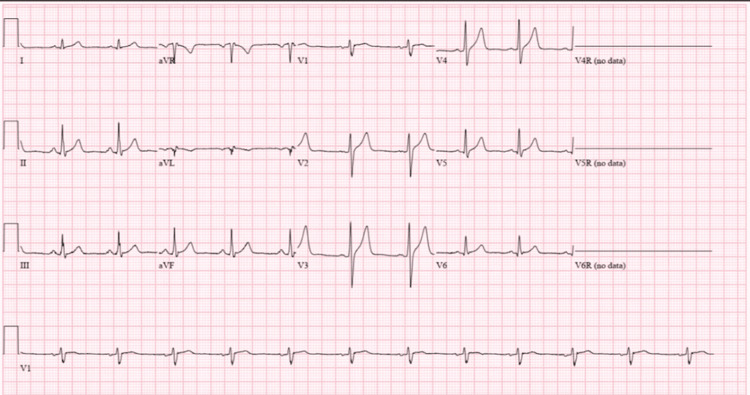
Resolution of the coved-shaped ST elevation that was seen in Fig. 1

The patient was started on full acute coronary syndrome (ACS) protocol and was given one dose of lorazepam 2 mg intravenously (IV) with the resolution of symptoms. The ECG was normal with a left ventricular ejection fraction of 60-65%. The cardiovascular stress test and nuclear imaging were negative for myocardial ischemia, hence the ACS protocol was discontinued. The next day, the Brugada pattern got resolved (Fig. 2), so the patient was discharged home in a hemodynamically stable condition with extensive counselling on the avoidance of cocaine.

## Discussion

It is no secret that the coronary-spastic effect of cocaine can lead to ischemia and dysrhythmia. As the leading cause of drug-related death in the United States, cocaine can lead to life-threatening cardiac complications such as sudden cardiac death, aortic dissection, stroke, and myocardial infarction [8]. Cocaine is a sympathomimetic stimulant that blocks serotonin, norepinephrine, and dopamine reuptake. These features increase the risk of ventricular irritability, leading to fibrillation. In addition, cocaine also has a potent sodium channel blocking effect, which can prolong QT and QRS intervals, inhibiting the generation of action potentials - like that of classIC antiarrhythmic agent flecainide [9]. Some cardiac dysrhythmias associated with cocaine use are complete bundle branch block, ventricular tachycardia, supraventricular tachycardia, sinus bradycardia, torsades de pointes, and Brugada pattern [10].

Brugada syndrome is a life-threatening cardiac arrhythmia characterized by an autosomal dominant sodium channel gene mutation in SCN5A. Accounting for 0.1% of the population, this syndrome is known for causing sudden cardiac death in young males [11]. The SCN5A gene is responsible for encoding alpha subunits of the myocardial sodium channels. These defective sodium channels reduce the inflow of sodium while increasing the outward current. This change in mechanism leads to a plateaued action potential and a vulnerability for reentry ventricular arrhythmia [12]. There are three ECG patterns for Brugada syndrome due to the SCN5A gene mutation. Type 1, also known as the Brugada sign, is a coved ST-segment elevation >2 mm in >1 of V1-V3 followed by a negative T wave. Type 2 shows >2 mm saddleback-shaped ST elevation with a positive T wave (terminal portion of ST segment). Lastly, type 3 can be the morphology of either type 1 or type 2, but with < 2 mm of ST-segment elevation and a positive T wave. Diagnosis of Brugada syndrome depends on characteristic ECG findings and clinical criteria. Clinical symptoms must include at least one of the following: documented ventricular fibrillation or polymorphic ventricular tachycardia, a family member of sudden cardiac death <45 years old, inducibility of ventricular tachycardia via programmed electrical stimulation, syncope, or nocturnal agonal respiration [13].

One of the unique features in our case was the transient Brugada pattern observed after cocaine intoxication. Brugada sign has been seen in asymptomatic patients after exposure to various drugs such as cocaine, lithium, tricyclic antidepressants, fluoxetine, trifluoperazine, and antihistamines [14]. Brugada ECG patterns are seen in these asymptomatic patients, who do not have a family history of Brugada syndrome due to the exposure to sodium channel blocking effects. It is unknown whether these patients need to have an underlying genetic predisposition to Brugada syndrome or if ECG patterns represent a latent Brugada syndrome [15].

Brugada syndrome should not be confused with other conditions which may mimic Brugada-like patterns. Normal hearts with electrolyte imbalance or hypothermia may show similar ECG findings. In addition, acquired and hereditary structural diseases such as pulmonary embolism and arrhythmogenic right ventricular cardiomyopathy, respectively, should not be confused either. These conditions all have different pathophysiologies and prognoses [13].

Although cocaine has no approved antidote, patients with Brugada syndrome are treated with standard, supportive therapy. This therapy includes nitroglycerin to alleviate vasospasm, benzodiazepine to counteract the sympathomimetic effect, correction of metabolic disturbance, and administration of antiarrhythmic agents [10]. Sodium bicarbonate has been seen to reduce cocaine-induced wide-complex tachycardia by alkalizing extracellular fluids, thereby disassociating cocaine from sodium channels [16]. Implantable cardioverter-defibrillator (ICD) implantation is necessary for patients with Type 1 ECG patterns and symptoms of syncope, seizure, and nocturnal agonal respiration [17].

## Conclusions

In conclusion, as a sodium channel blocker, cocaine may unmask latent Brugada syndrome in asymptomatic patients without a family history. Recognizing Brugada syndrome on ECG is vital to avoid misdiagnosis and mistreatment of the patient with or without a genetic predisposition. However, it is still unknown if drug-induced or latent Brugada syndrome has the same complications as full-blown Brugada syndrome.

## References

[1] (2019). Centers for Disease Control and Prevention. Multiple Causes of Death 1999-2017 on CDC WONDER. https://wonder.cdc.gov/mcd.html.

[2] McCord J, Jneid H, Hollander JE (2008). Management of cocaine-associated chest pain and myocardial infarction: a scientific statement from the American Heart Association Acute Cardiac Care Committee of the Council on Clinical Cardiology. Circulation.

[3] Benzaquen BS, Cohen V, Eisenberg MJ (2001). Effects of cocaine on the coronary arteries. Am Heart J.

[4] Starmer CF, Grant AO, Strauss HC (1984). Mechanisms of use-dependent block of sodium channels in excitable membranes by local anesthetics. Biophys J.

[5] Singh N, Singh HK, Singh PP, Khan IA (2001). Cocaine-induced torsades de pointes in idiopathic long Q-T syndrome. Am J Therapeutics.

[6] Tomcsányi J (2016). [Brugada phenocopy]. Orv Hetil.

[7] Xu G, Gottschalk BH, Anselm DD (2018). Relation of the Brugada phenocopy to hyperkalemia (from the International Registry on Brugada Phenocopy). Am J Cardiol.

[8] Mittleman RE, Wetli CV (1984). Death caused by recreational cocaine use. An update. JAMA.

[9] Bauman JL, Grawe JJ, Winecoff AP, Hariman RJ (1994). Cocaine-related sudden cardiac death: a hypothesis correlating basic science and clinical observations. J Clin Pharmacol.

[10] Lange RA, Hillis LD (2001). Cardiovascular complications of cocaine use. N Engl J Med.

[11] Fowler SJ, Priori SG (2009). Clinical spectrum of patients with a Brugada ECG. Curr Opin Cardiol.

[12] Raharjo SB, Maulana R, Maghfirah I, Alzahra F, Putrinarita AD, Hanafy DA, Yuniadi Y (2018). SCN5A gene mutations and the risk of ventricular fibrillation and syncope in Brugada syndrome patients: A meta-analysis. J Arrhythm.

[13] Antzelevitch C, Brugada P, Borggrefe M (2005). Brugada syndrome: report of the second consensus conference: endorsed by the Heart Rhythm Society and the European Heart Rhythm Association. Circulation.

[14] Yap YG, Behr ER, Camm AJ (2009). Drug-induced Brugada syndrome. Europace.

[15] Hermida JS, Jandaud S, Lemoine JL (2004). Prevalence of drug-induced electrocardiographic pattern of the Brugada syndrome in a healthy population. Am J Cardiol.

[16] Wang RY (1999). pH-dependent cocaine-induced cardiotoxicity. Am J Emerg Med.

[17] Olde Nordkamp LR, Wilde AA, Tijssen JG, Knops RE, van Dessel PF, de Groot JR (2013). The ICD for primary prevention in patients with inherited cardiac diseases: indications, use, and outcome: a comparison with secondary prevention. Circ Arrhythm Electrophysiol.

